# The roles of pets in long-term care at home: a qualitative study

**DOI:** 10.1186/s12877-023-04416-w

**Published:** 2023-10-30

**Authors:** Peter W.A. Reniers, R. Leontjevas, I. J.N. Declercq, M-J. Enders-Slegers, D. L. Gerritsen, K. Hediger

**Affiliations:** 1 Faculty of Psychology, Open University of the Netherlands, Heerlen, The Netherlands; 2 Department of Primary and Community Care, Radboud University Medical Centre, Radboud Institute for Health Sciences, Radboudumc Alzheimer Centre, Nijmegen, The Netherlands; 3 Faculty of Psychology, University of Basel, Basel, Switzerland

**Keywords:** Home care, Community care, Long-term care, Pets, Older adults

## Abstract

**Background:**

Pets play very important roles for older adults. However, whether the same roles apply to pets of care clients receiving long-term care at home (LTCH) is unclear. This study aimed primarily to explore whether the roles of pets for LTCH-clients who own pets are comparable to the roles of pets for older adults in the general population. Furthermore, we explored potential pet-related problems that might be encountered in LTCH in practice, and the potential influences of pet ownership on caregiving relationships. These insights may help improve long-term care services in LTCH.

**Methods:**

This project started with a study using the Consensual Qualitative Research method (CQR). We conducted semi-structured interviews based on themes from our previous review (e.g., Relational Aspects, Emotional Aspects, and Social Aspects). Secondly, an online survey was used to confirm the findings from the CQR study by calculating Content Validity Index scores (in SPSS 26) regarding contents, relevance, and clarity. The survey also included open-ended questions on potential pet-related problems and their impact on caregiving relationships for LTCH-clients, family caregivers, and professional caregivers.

**Results:**

The CQR study found that the roles pets play for LTCH-clients (N = 8), family caregivers (N = 10), and professional caregivers (N = 10) were similar to the roles pets play for older adults in the general population. The online survey confirmed most of the CQR findings. In the survey, LTCH-clients (N = 4), family caregivers (N = 8), professional caregivers (N = 8), and researchers in human-animal studies and in geriatric care (N = 5) reported various potential problems that could arise from pet ownership by LTCH-clients, such as clients with deteriorating health being forced to part with their pets. Participants also reported potential positive and negative effects of pet ownership on caregiving relationships, such as pets being a nice topic of conversation, or, conversely, a source of disagreement in the LTCH context.

**Conclusions:**

The roles pets play for LTCH-clients seem comparable to the roles pets play for older adults in the general population. In addition, LTCH-clients might experience pet-related problems specific to the LTCH context. Pets may influence caregiving relationships, either positively or negatively. Therefore, instruments and guidelines are needed to account for pets in LTCH.

**Supplementary Information:**

The online version contains supplementary material available at 10.1186/s12877-023-04416-w.

## Introduction

Pets play various roles in peoples’ lives. In our previous review of qualitative studies on pets’ roles in the lives of older adults, we identified several different roles played by pets: friend, family member, facilitator of social connections, and provider of emotional support, company, physical contact, sense of safety, and meaning [1]. These roles indicate the importance of pets to many people, and their centrality in people’s lives shows that pets can have an impact on an owner’s wellbeing.

As a consequence of the growing number of older adults requiring health care [2], Western countries are now promoting long-term care at home (LTCH) over institutionalised care. As a result, an increasing number of people with chronic illness, and people with physical, mental, somatic, or sensory disabilities or psychogeriatric illnesses now receive LTCH [3]. Considering that over half of all households in Western countries own pets [4–6], many LTCH-clients are also likely to be pet owners. Through the various roles they play, pets may influence the lives and wellbeing of LTCH-clients as well.

To the best of our knowledge, no studies to date have reported on the prevalence of pet ownership in the LTCH context and there remains a dearth of research on pets’ roles for LTCH-clients. Some evidence indicates that LTCH-clients experience the roles of pets in much the same way older adults in the general population experience them [7, 8]. Nonetheless, there may be important differences between these two groups, particularly in how the care needs of LTCH-clients impact others. First, LTCH-clients may need help from others to care for their pets. This task often falls on family caregivers, which can result in additional burden for the caregiver [9, 10]. Since being a family caregiver is associated with an increased risk of burnout [11, 12], additional burden can potentially cause serious health issues for family caregivers and influence the care they are able to provide. Second, pets may impact the professional caregiving practice—for instance, a professional caregiver may be afraid, allergic, or not fond of animals. This may negatively impact the professional caregiver as well, because caregiving tasks may have to be performed while the caregiver is experiencing severe anxiety [13]. Consequently, it may negatively influence the provided quality of care, and may have an adverse impact on the client. Moreover, it can negatively impact LTCH-clients, since rejection of the pet by others may have an impact on an owner’s self-esteem, and feelings of belonging, control, and meaningful existence for owners [14]. Third, owning a pet may influence healthcare-related decisions. Some pet owners may delay medical treatment or institutionalisation due to concerns about their animal. This is especially true for people who have a small social network and limited resources to care for their pet [15]. When this occurs, pet ownership may negatively impact the health of pet owners.

Pet ownership by LTCH-clients may have positive or negative impacts not only for LTCH clients, but also for others, including family caregivers and professional caregivers. Thus, it is important to investigate what roles pets play for LTCH-clients and to explore the impact of pets on those who provide care in an LTCH context. For these reasons, the current study was primarily aimed at exploring whether the roles pets play in the lives of LTCH-clients are comparable to the previously identified roles they play for older adults in the general population. Furthermore, we explored potential problems related to pet ownership that may be encountered in the LTCH-practice and the potential influence of pet ownership by LTCH-clients on caregiving relationships within the triad of the LTCH-client, family caregivers, and professional caregivers.

## Method

### Research design

We used several methods that included the Consensual Qualitative Research (CQR) method to explore the different roles of pets in LTCH. In addition, we used an online survey to determine the content validity (CV) of the CQR findings and explore the potential problems and the potential influence of pet ownership in LTCH on caregiving relationships. Mixed Method Appraisal Tool criteria were considered while drawing up the final version of the manuscript [16, 17].

### Study procedures

#### Participants and recruitment

Dutch speaking LTCH-clients with pets, their family caregivers, and professional caregivers were eligible to participate. For both the CQR-method and the online survey, participants were recruited through two community-care organisations and an organisation that supports family caregivers, all active in the south-eastern part of the Netherlands. The community-care organisations distributed an information letter to LTCH-clients with pets. The family caregiver organisation sent the information letter to family caregivers who had given their consent to be contacted about ongoing research. In addition to LTCH-clients, their family caregivers, and professional caregivers (the caregiving triad), experts in human-animal studies and in geriatric care were included in the online survey. These experts were contacted through the research group members’ own professional networks. Participation in one or both studies was allowed.

LTCH-clients, family caregivers, and professional caregivers who participated during their leisure time were offered a 20-Euro gift. Employees of one of the organisations could participate in the study during their working hours.

### Materials and procedures

#### Consensual qualitative research interview protocol

The CQR-method [18, 19] involved multiple analysts and auditors. There were seven steps: (1) Developing an interview protocol; (2) Conducting and transcribing semi-structured interviews; (3) Analysing topics and ideas from individual transcripts; (4) Exploring core ideas from individual transcripts; (5) Auditing the topics and core ideas (Steps 3 and 4); (6) Conducting cross-analyses (categorisation of topics across all transcripts); (7) Auditing the cross-analyses [18, 19]. Alongside an inductive approach, as prescribed by the CQR-method, we applied a deductive approach in step 6 based on the seven themes from our previous review [1] describing the various roles of pets for older adults in the general population. The CQR-method has a focus on reaching consensus amongst team members through regular rounds of discussion while also allowing for reflexivity [18–20].

The interview protocol included brief explanations of the various roles that pets play for older adults clustered into seven themes, namely: Relational Aspects, Reflection and Meaning, Emotional Aspects, Aspects of Caregiving, Physical Health, Social Aspects, and Bidirectional Behaviour [1]. The explanations and prompts were refined during research team working group discussions. Examples of prompts were *‘Do you recognise the description of this theme?’* and *‘Is there a difference between before when you did not need care and now? Can you give several examples?’*. Participants could indicate if they preferred to be interviewed face-to-face, via Microsoft Teams, or by telephone. Prior to the interviews, all participants provided informed consent. Interviews took place between 4 and 2021 and 11 November 2021 and lasted between 22 and 117 min. LTCH-clients and family caregivers could choose to be interviewed together. The interviews were transcribed verbatim. See Appendix A1 for the full interview protocol.

#### Online survey

Participants rated specific roles of pets that emerged from the CQR-method regarding the content, relevance, and clarity of statements on a four-point scale which was designed based on the recommendations of Polit and Beck [21]. For instance, one of the questions after the description of a role was *‘Do you think it is relevant?’.* Participants rated the questions with *‘Not at all’*, *‘Somewhat’*, *‘Quite’*, and ‘*Completely*’. Every participant rated three individual themes. In order to match themes to participants, we used random numbers generated automatically.

The survey had additional open questions on potential problems that could arise from pet ownership in LTCH as well as potential influences on caregiving relationships (e.g., between a LTCH-client and family caregiver). Data were collected between 14 March 2022 and 18 April 2022. The survey for a single theme is added as Appendix A2.

### Analyses

#### Consensual qualitative research analysis

The research team consisted of two PhD students (PR and ID), two experts in human-animal studies (KH and ME), and two experts in geriatric care research (DG and RL). A specialist in geriatric medicine (LS), and an MSc psychology intern (MM) were also involved in the research and recruitment process.

The interview transcripts were analysed in ATLAS.ti 9 for Windows. Initially, three analysts (PR, ID, and MM) individually applied open coding and an inductive iterative approach uncovering topics and core ideas within single interviews. This part of the analysis was audited by four experts (ME, DG, RL, and LS). Subsequently, using a deductive approach, the initial codes were categorised by the same analysts using the seven themes from our previous review in the general population of older adults [1]. This part of the analysis was audited by two experts (DG and LS).

The outcomes were summarised on theme cards representing the main themes. The cards included citations to reflect the perspectives of different stakeholders. The cards were used as input for the online survey.

### Online survey analysis

We calculated Item Content Validity Indexes (CVI’s) for specific roles and Scale-CVIs for the seven themes in SPSS 26 [22] to determine the content validity (CV) of the CQR findings [21]. The indices reflected contents (*Do you recognise this [statement]?*), relevance (*Do you think this [statement] is relevant?*), and clarity (*Do you think this [statement] is clear?).*

Item-CVIs were calculated as follows: first, the four-point scale was dichotomised *‘Not at all’* and *‘Somewhat’* were scored 0, and *‘Quite’* and *‘Completely’* were scored 1. Subsequently, the Item-CVI score was calculated by adding up the scores of the raters and then dividing it by the total number of raters [21]. The Item-CVI, in case there were more than five raters, should have been at least 0.78 [21].

Furthermore, the Scale-CVIs were calculated for the themes. These were calculated by dividing the sum score of all roles in a theme (i.e., sum of Item-CVI scores in a theme) by the total number of ratings [23]. For a minimum Scale-CVI the score should be at least 0.90 [21]. The open questions were analysed using structured tabular thematic analysis [23].

## Results

### Participant characteristics

For the CQR study, a total of N = 23 interviews were conducted (N = 19 by PR and N = 4 by ID) with 28 participants (Table 1). Ten interviews took place face-to-face, eleven via Microsoft Teams, and two by telephone. In five of the interviews, both a client and a family caregiver were interviewed together. With the exception of two participants, one who owned a cat and the other who owned a rabbit, all of the LTCH-client and family caregiver participants owned dogs. In the online survey, a total of N = 25 participants provided their response (Table 2).

**Table 1:** CQR Participant characteristics

	Gender	Age	Pet	Pet Age	
**Clients**:					
CL 1	Male	16	Dog	1	
CL 2	Female	77	Dog	10	
CL 3	Female	47	Rabbit	3	
CL 4	Male	73	Dog	8	
CL 5	Female	85	Dog	11	
CL 6	Female	72	Cats	(deceased)	
CL 7	Male	64	Dog	7	
CL 8	Male	82	Cat	1	
**Family Caregivers**:					**Care recipient (not interviewed) Gender (Age)**
IC 1	Female	63	Dog	16	Female (93)
IC 2	Female	75	Two dogs	10 and 11	Male (77)
IC 3	Female	73	Dog	6	Male (64)
IC 4	Female	60	Dog	8 months	Male (65)
IC 5	Female	59	Dog	10	Male (62)
				**Interviewed together with client**	
IC 6	Female	49		CL1	
IC 7	Female	49		CL2	
IC 8	Male	56		CL3	
IC 9	Female	71		CL4	
IC 10	Male	84		CL5	
**Caregiving Professionals**:			**Function**	**Work Experience (years)**	
CP 1	Female	45	Caretaker	25	
CP 2	Female	34	Nurse	11	
CP 3	Female	55	Caretaker	47	
CP 4	Female	23	Nurse	2	
CP 5	Female	55	Nurse	36	
CP 6	Female	63	Case manager	35	
CP 7	Female	33	Nurse	13	
CP 8	Female	52	Nurse	26	
CP 9	Female	62	Nurse Practitioner	44	
CP 10	Female	42	Nurse	22	

**Table 2:** Online Survey Participant Characteristics

Participants		Gender	Age Range	Level of Education 1/2/3
Clients	N = 4	Male = 0 Female = 4	58–72	3/1/0
Family Caregivers	N = 9	Male = 2 Female = 6	44–71	2/4/3
Caregiving Professionals	N = 7	Male = 0 Female = 7	23–55	2/3/2
Subject Matter Experts	N = 5	Male = 0 Female = 5	24–46	0/0/5

N. The level of education is listed as 1/2/3; Secondary General Education/Higher Professional Education/University. In the table column the numbers represent the N.

### Roles of pets in the lives of LTCH-clients

The outcomes of the CQR-method corresponded with the roles found in the previously conducted review. The themes were: Relational Aspects, which describes roles related to the bond between an owner and the pet (e.g., the pet as an attachment figure); Reflection and Meaning, which relates to certain beliefs and convictions of the pet owner (e.g., the pet as a source of meaning to the owner’s life); Emotional Aspects, which is characterised by roles related to feelings (e.g., the pet provides emotional support); Aspects of Caregiving, which relates to caring for and worries about the pet (e.g., pet related worries); Physical Health, which contains roles that can influence an owner’s health (e.g., additional exercise from performing pet-related chores such as walking the dog); Social Aspects, which depicts roles related to meeting other people and loneliness (e.g., the pet facilitates social connection to others), and Bidirectional Behaviour, which describes roles related to the pet’s physical presence (e.g., hugging the pet). However, no codes were found that corresponded with the review’s role Medical Detection. In this role, pets might notice illnesses or upcoming seizures in their owners and react to or warn of these events [1]. Therefore, this role was not used in further steps of this study. The roles found in the CQR analyses and their CVI’s are presented in the Appendix (see Appendices A3 to A9). Most of the roles were confirmed by sufficiently high CVIs regarding contents, relevance, and clarity.

The exceptions with a CVI score below the cut-off points were the Item-CVI for relevance for the role Grief (Theme Emotional Aspects, Appendix A5). The Item-CVIs for content for the roles Sense of Safety and Expenses which corresponded to a too-low contents Scale-CVI for the theme Aspects of Caregiving (Appendix A6). Furthermore, the role Mirroring scored too low on contents, relevance, and clarity resulting in too-low Scale-CVIs for content and clarity for the theme Bidirectional Behaviour (Bidirectional Behaviour, Appendix A9).

### Potential pet-related problems

The answers regarding potential problems were summarised in five topics, namely: (1) LTCH-client health deterioration may complicate the pet’s care: (2) ethical issues regarding pets (e.g., poor pet care or an unsafe pet environment); (3) forced pet relinquishment (e.g., due to moving to a nursing home); (4) pet bereavement; and (5) the family (pet) caregiver is not available to aid in pet care (e.g., due to hospital admission).

1. *LTCH-client, female, 66 y.o.: ‘I am a pulmonary patient and there will come a time when I cannot walk my doggie anymore’.*
2. *Professional caregiver, female, 40 y.o.: ‘For a pet it is important that it is kept in a safe environment with enough room to fulfil its needs… If someone has difficulty reading or taking care of [a pet] then that is not guaranteed’.*
3. *Subject matter expert, female, 24 y.o. ‘When a pet is very important to a client, but their health does not allow it anymore. Then they would be forced to relinquish their pet which could cause depression and loneliness. It was not the client’s choice’.*
4. *Professional caregiver, female, 40 y.o.: ‘When a pet dies it can be very disheartening and disrupting [for a client]’.*
5. *Family caregiver, male, 71 y.o.: ‘As a family caregiver I have been out of running for a while. Walking the dog is a problem then’.*

### Influence of pets on caregiving relationships

When asked about the influence of pets on caregiving relationships, participants gave examples of potential positive and negative influences. These could be due either to a pet’s impact on the LTHC atmosphere (i.e., creating a pleasant or an unpleasant atmosphere) or a positive or negative conversation topic (i.e., nice to talk about or a reason to argue). The influences of pets on caregiving relationships could be summarised in two positive and two negative points. The positives were: (1) pets create a pleasant atmosphere; and (2) pets are a nice topic of conversation; The two negative points were: (3) pets can be frustrating during caregiving; and (4) differences in opinion between stakeholders related to pet care.

1. *Professional caregiver, female, 26 y.o. ‘When a client feels better because of a pet, this has benefits for the family caregiver and the professional care worker as well’.*
2. *Family caregiver, male, 57 y.o.: ‘A pet is a nice topic of conversation’.*
3. *Subject matter expert, Female, 37 y.o.: ‘A pet can be too intrusive, cost too much energy, be in the way during caregiving etcetera’.*
4. *Family caregiver, male, 70 y.o.: ‘Arguments or friction about how a pet is (unintentionally) treated’.*

## Discussion

This study revealed that, according to caregiving triad representatives, the roles played by pets for clients in long-term care at home (LTCH) were similar to the roles played by pets for older adults in the general population. Moreover, five clusters of potential problems were reported for pet ownership in the LTCH-setting. The potential influence of pets on caregiving relationships within the caregiving triad could be experienced either positively or negatively.

Overall, participants indicated that their pets were important to them and contributed to their wellbeing. However, (quantitative) scientific research investigating the relationship between pet ownership and wellbeing is incongruent (e.g., [24–26]). Nonetheless, some evidence suggests a positive relationship between a higher quality of attachment to a pet and its owner’s wellbeing [24]. People who have an insecure attachment to their pet are more likely to experience psychological distress and lower wellbeing [24]. This suggests that supporting the attachment between an LTCH-client and a pet is important, which could possibly be achieved by making plans concerning sustainable pet care with LTCH-clients.

There were some inconsistencies between the outcomes of the interviews and the online survey. Participants did not rate the role of pet-related grief under emotional aspects as relevant for LTCH-clients. It is plausible that participants perceive the death of a pet as a natural part of life, thus considering it as an inherent aspect of pet ownership. However, pet bereavement was mentioned as a potential problem. Research indicates that pet bereavement can lead to high levels of grief for long periods (weeks to months), loss of social contacts (e.g., while walking the dog), and loss of relationship and support experienced from the pet [27]. Thus, pet bereavement can negatively impact several domains in clients’ lives, and, therefore, requires the attention of healthcare services to improve wellbeing for LTCH-clients—for instance, through supportive counselling.

Pet relinquishment may also lead to grief. A study that looked into the reasons why pet owners relinquished their pets (dogs and cats) to a Danish animal shelter over the course of 20 years found that the most common reasons for pet relinquishment were an owner’s health (31% overall) and issues with housing such as regulations about pets in residential care (23% overall) [28]. Anticipating pet relinquishment and its accompanying grief may lead some LTCH-clients to delay seeking health care (e.g., admission to a nursing home) [15]. This delay can have a negative impact on the LTCH-client’s health and subsequently increase the risk of further health deterioration and healthcare costs.

When LTCH-clients experience deteriorating health, they may come to rely more on family caregivers to provide care for their pets. Despite the potential benefits (e.g., emotional support) of a pet’s presence, family caregivers may not have sufficient time to help care for the pet. They may experience caring for a pet as a burden [29]. Sometimes pet care becomes the exclusive responsibility of the family caregiver. According to participants in our study this can become problematic—for instance, if the family caregiver becomes unavailable to care for the pet (e.g., due to their own hospitalisation). Therefore, it may be useful for pet owners receiving LTCH to make arrangements with others beforehand to support in pet caregiving.

The pet’s role Mirroring found in the previous review was not recognised by the participants in this study. Mirroring, however, is an important aspect of some animal-assisted therapies [30]. In equine-assisted therapy, for example, therapists use the horse’s ability to respond to, or mirror, the inner emotions and intentions of people. When a client is worried and experiences anxiety, this can be observed in the worried and anxious behaviour of the horse [30]. Mirroring can be a useful way to help clients explore and regulate their emotions [31]. Some evidence shows that dogs also mirror their owners’ stress levels [32]. A possible explanation for the finding in our study is that mirroring is easier to detect in therapeutic and research settings than in daily interactions between pet owners and their pets.

The content of the role Pet-Related Expenses on pet caregiving was not recognised in our study and pet costs were not reported as a potential problem. The participants in this study may have had sufficient funds to care for their pet; alternatively, they might have been reluctant to discuss existing financial problems. However, participants did find pet-related expenses worth considering. Expenses related to pet ownership can be problematic for people on limited budgets and for those with physical limitations [33]. Therefore, it may be useful for professional caregivers to make their LTCH-clients on a limited budget aware of pet-related resources such as local pet food banks.

Overall, several of the potential problems participants reported can be anticipated and solutions found prior to their becoming actual problems. Hence, increasing awareness of and information exchange about potential pet-related problems and solutions within the caregiving triad may be particularly useful. This could take place, for instance, during care planning talks.

Remarkably, participants did not mention an increased risk of falls as a potential problem of pet-ownership, despite research indicating that pets are a major fall risk to older adults which can cause serious injuries [34, 35]. For instance, people can fall over their pet’s toys or the pet itself, or they can trip, slip, or stumble while walking a dog. These types of accidents can substantially impact an owner’s health [34–36]. The study participants may not have reported falls as a potential problem because they may never have witnessed or experienced pet-related falls themselves. Falls resulting from pet-related clutter or interactions could pose a concern, particularly to LTCH-clients living without a partner or those lacking assistance with household chores. Healthcare organisations can play a role in preventing falls by creating awareness about a safe home environment—for instance, pet items should not be placed in walkways and it is important that rooms be well-lit when walking around [37].

In addition to the positive influences of pets on caregiving relationships, such as pets being a pleasant topic of conversation, participants also reported two potential negative influences of pets. Problems can arise when pets are in the vicinity while LTCH-clients are receiving care. For example, a dog might try to intervene to protect its owner. In such a situation, the pet may need to be put in another room during caregiving [7], which may negatively impact the caregiving relationship between the LTCH-client and the professional caregiver. Differences in opinion related to pet care may also cause friction within the care relationship. For instance, professional caregivers expressing pet care concerns may seem meddlesome to LTCH-clients and their family caregivers. Additionally, family caregivers who experience burden may project their frustration onto the care recipient. For example, if they are unable to plan personal days due to pet care duties, have a dislike for the pet, or suffer from pet allergies themselves. The potential for these sorts of problems and the impact they can have on caregiving relationships suggest that pets need to be accounted for in the LTCH-setting.

### Strengths, limitations, and Future Research

Strengths of this study include its use of several rigorous methods, multiple analysts and auditors, and several rounds of working group discussions. A limitation is that most of the people we interviewed were dog owners who were overall very positive about owning a pet. A second limitation pertains to interviews conducted jointly with both the client and family caregiver. It must be acknowledged that this approach may have influenced the responses provided, although the interviewers did not notice any constraints during the interviews. Future research should look further into the potential problems and influences of pet ownership on caregiving relationships, preferably using longitudinal and mixed methods designs. Determining which pet-related problems most urgently need to be solved should also be a priority. It is important to involve stakeholders of the caregiving triad (pet-owning LTCH-clients, family caregivers, and professional caregivers) and experts of various backgrounds.

### Practical implications

Conversations about pets between LTCH-clients, family caregivers, and professional caregivers may help each stakeholder anticipate potential problems and lead to satisfactory arrangements for pets. Preventing and solving pet-related problems is important for each stakeholder within the caregiving triad. Arrangements and relevant information concerning potential difficulties could be registered in a care plan. For example, this might include recording an address for (permanent or temporary) pet care in case the owner is hospitalised, or requesting additional support from an LTCH-client’s family members, neighbours, or volunteers in caring for a pet. Furthermore, professional caregivers may be able to improve the caregiving relationship with LTCH-clients and family caregivers through the attention they pay to their clients’ pets. Insight into the positive and negative influences pets have on caregiving relationships can be used to further improve relationships between pet-owning LTCH-clients, family caregivers, and professional caregivers. Attention and support from healthcare organisations with the use of tools and guidelines that account for the different roles of pets may improve the wellbeing of all those involved.

## Conclusions

This study provided useful information related to pets in the context of long-term care at home. Older adults in LTCH seem to perceive the roles of pets in much the same way that older adults in the general population do [1]. However, the potential problems of pet ownership by LTCH-clients reported in our study, such as the client’s health deterioration, reliance on family caregivers to care for pets, and the positive and negative impact of pets on caregiving relationships, require the attention of healthcare professionals.

### Electronic supplementary material

Below is the link to the electronic supplementary material.

Supplementary Material 1

## Data Availability

Data is available from the corresponding author on reasonable request.

## Abbreviations

LTCH: long-term care at home
CQR: consensual qualitative research
CV: content validity
CVI: content validity index
