# Supplementary material for: The roles of pets in long-term care at home: a qualitative study
Source: BMC Geriatr. 2023 Oct 30;23:702. doi: 10.1186/s12877-023-04416-w (PMC10617039; doi:10.1186/s12877-023-04416-w)
Supplement: Supplementary file 1 — Supplementary Material 1 [file 12877_2023_4416_MOESM1_ESM.docx]

**Appendices**

**Appendix A1**

*Interview protocol*

*Themes:*

**Relationship with the pet:** This is the bond with a pet. People do not have to act differently for their pet, the pet does not set conditions for its affection, and their pet does not judge. Also, many people notice that their pet cares about them.

**Feelings and emotions:** This is about feelings and emotions in relation to pets. People can derive a great deal of pleasure from their pets. Additionally, pets react to the feelings of their owners and other people. Pets can provide emotional support, for instance, when someone is sad. Owners often miss their pets deeply if they are forced to let them go or if the pet passes away.

**Caring for and worries about pets:** Many people feel a need to care for something. It can be nice to be responsible for another being. Pets may also take care of people in their own way—for instance, a pet may create a safer environment for its owner. But pets can also be a source of worry. For example, owners often worry about their pet’s health. Moreover, pet ownership involves expenses for things like food and veterinary care.

**Physical Health:** Pet ownership often involves additional physical activity, which can have a positive effect on a pet owner’s health. Pets can also provide structure to an owner’s day, be a source of relaxation for owners, and distract from discomfort or pain.

**Social contacts:** Pets help reduce feelings of loneliness and owning a pet can facilitate contact with other people. For example, a dog owner might meet people while walking the dog, or a cat owner might join a Facebook group for cat enthusiasts.

**Presence:** This relates to the physical presence of the pet. Pets also seek their owner’s proximity. Touching, such as petting and hugging, can be enjoyed by both people and pets. Pets also react to the behaviour and environment of their owners.

**Thoughts in the Present and Past:** People recognise different emotions in their pets. A person can often recognise when their pet is happy, sad, or jealous. Some people also have entire conversations with their pets and believe that their pets understand them. Pets can also evoke memories about the past. Some people say that their pet gives their life a purpose.

*Prompts for clients and family caregivers:*

- Do you recognise the description of this theme? How is that for you? Can you provide examples?
- Is there a difference between before when you did not need care and now? Can you provide examples?
- To what extent do other people play a role in this (e.g., caregivers and loved ones)? Can you give examples? How is it for you, and for them?

*Prompts for professional caregivers:*

- Do you see this theme reflected in the clients you provide care to? What do you think of that? Can you provide examples?
- What does this theme mean to you personally, and do you take it into account in your work? Can you give examples? How does the client appreciate that?

**Appendix A2**

*Example of part of the online survey*

**Relationship with the pet - Text**

In previous research, we found several themes relating to experiences with pets such as Physical Health and Emotions.

The themes are briefly described on different cards. We also summarised what clients, family caregivers, and professional caregivers said about a theme.

The card on this page is about the relationship between an owner and a pet. It is about attachment (the relationship), unconditional love, and interdependence.

We would like to know your opinion on each theme. Below is a picture of the card. You do not have to read it in detail because after the picture the themes will be presented to you separately.

Would you rate the statements on content, relevance, and clarity?

|  | Not at all | Somewhat | Quite | Completely |
| --- | --- | --- | --- | --- |
| Do you recognise this? |  |  |  |  |
| Do you think it is relevant? |  |  |  |  |
| Do you think it is clear? |  |  |  |  |

| Could you explain why you gave the scores above? |
| --- |
|  |

The following questions are about the contents of the card about the relationship with the pet. Below is a picture of the entire card. You have already read most of it on the page before.

| Can problems occur due to the subjects discussed on this card? For example, now, in the future, or due to a client’s health deteriorating. | | |
| --- | --- | --- |
| Yes | No | I don’t know |
|  |  |  |

| Could you explain why you gave this answer? |
| --- |
|  |

| Can the discussed subjects influence relationships between clients, family caregivers, and professional caregivers? | | |
| --- | --- | --- |
| Yes | No | I don’t know |
|  |  |  |

| Could you explain why you gave this answer? |
| --- |
|  |

**Appendix A3**

*Relational Aspects*

| **Front: Themes** | | **Back: Stakeholder Perspectives** | |
| --- | --- | --- | --- |
| **Attachment:**   - Owners experience strong bonds with their pets. - Owners view their pet as a friend and part of the family. - Owners involve their pets in different activities. - Owners find it difficult when they have to say farewell to their pet. | | **Clients say:**   - My pet is my friend. - I notice that my pet loves me also. - I believe my pet accepts me for who I am. - I eat a bit less for a few weeks so I can save money to go to the vet. | |
| **Unconditional Love:**   - Owners experience their pet’s love as unconditional. - A pet does not ask difficult questions, does not judge, keeps a secret, and is always available. - Owners can be themselves with their pets. | | **Family caregivers say:**   - My partner has a strong bond with our pet. - The bond with the pet means a lot to my partner. - I have a strong bond with our pet as well. | |
| **Interdependence:**   - Owners take care of their pets, but the pet also takes care of its owner. - A pet can help its owner through difficult situations. - A pet can fill feelings of emptiness and help the owner feel well. | | **Professional caregivers say:**   - I often see a very strong bond between clients and their pets. - I notice that the bond with a pet is especially strong when the client is the pet’s main caretaker. - Clients who deteriorate can experience anxiety related to their pet. - Pets trust their owners. - A pet offers feelings of security to clients. | |
| **N = 8** | **Content** | **Relevance** | **Formulation** |
| **Attachment** | Item-CVI = 1.00 | Item-CVI = 1.00 | Item-CVI = 1.00 |
| **Unconditional Love** | Item-CVI = 1.00 | Item-CVI = 1.00 | Item-CVI = 1.00 |
| **Interdependence** | Item-CVI = 1.00 | Item-CVI = 1.00 | Item-CVI = 1.00 |
| **Relational Aspects** | Scale-CVI = 1.00 | Scale-CVI = 1.00 | Scale-CVI = 1.00 |

*Note.* The statements are translated from Dutch.

**Appendix A4**

*Reflection and Meaning*

| **Front: Themes** | | **Back: Stakeholder Perspectives** | |
| --- | --- | --- | --- |
| **Attribution of Feelings:**   - Every pet has his or her own personality. - Owners recognise different kinds of emotions in their pets. - Owners believe that their pets understand them. - Pets enthusiastically greet their owners when they have been away. | | **Clients say:**   - I have more time than I had before. Therefore, I spend more time on my current pet than I did on former pets. - I enjoy it when someone else gives my pet attention. - I feel that I am still useful because I have to care for my pet. - I have fond memories of my (former) pets, which I like to talk about. - I commemorate my former pets on the anniversary of their death. | |
| **Memories:**   - Owners have many memories of their pets. - Owners reminisce and talk about pets they owned before. - Owners place pictures of their pet in their homes as mementos of a deceased pet. | | **Family caregivers say:**   - A pet can make my life more bearable. - A pet gives substance to my life and serves as a pleasant activity. - I notice that my partner feels that he is involved in the household when he can help care for the pet. - I see that my brother starts to neglect himself when he does not have a pet. | |
| **Sense of Achievement:**   - Owners feel that taking care of a pet is a pleasant and useful activity. - Owners feel as if they belong with the pet. - A pet can have a positive influence on the self-esteem and self-respect of the owner. | | **Professional caregivers say:**   - I often notice that a pet is everything for a client. - Clients feel useful because of their pets, and this has a positive impact on their self-esteem. - Some clients say that they would not want to live anymore if they did not have their pet. | |
| **Meaning of Life:**   - Owners feel as if they need and foremost want to be there for their pet. - Pets give substance to the owner’s life. - Some owners say that they do not want to live without a pet. | |  |  |
| **N = 11** | **Content** | **Relevance** | **Formulation** |
| **Attribution of Feelings** | Item-CVI = 1.00 | Item-CVI = 1.00 | Item-CVI = 1.00 |
| **Memories** | Item-CVI = .91 | Item-CVI = .91 | Item-CVI = 1.00 |
| **Sense of Achievement** | Item-CVI = 1.00 | Item-CVI = 1.00 | Item-CVI = 1.00 |
| **Meaning of Life** | Item-CVI = 1.00 | Item-CVI = 1.00 | Item-CVI = 1.00 |
| **Reflection and Meaning** | Scale-CVI = .98 | Scale-CVI = .98 | Scale-CVI = .98 |

*Note.* The statements are translated from Dutch.

**Appendix A5**

*Emotional Aspects*

| **Front: Themes** | | **Back: Stakeholder Perspectives** | |
| --- | --- | --- | --- |
| **Responsiveness to Feelings:**   - Owners and pets respond to each other’s feelings. - A pet’s happiness is contagious: owners become happier themselves. | | **Clients say:**   - My pet notices when I am not feeling well. - My pet wants my attention, and this helps me feel better. - I enjoy it when I notice that my pet is happy. - The death of my pet was a terrible experience. - After my pet’s death I mostly miss the company and I want a new pet as soon as possible. | |
| **Emotional Support:**   - A pet provides its owners a lot of emotional support. - Owners say that they feel that their pets comfort them when they are not feeling well. - Pets fill a feeling of emptiness and provide support**.** | | **Family caregivers say:**   - The pet senses what mood I am in. - The pet helps me feel happier again sooner. - I can clear my mind when I walk the dog. - I enjoy walking the dog together with my partner who needs care. - I notice that the pet understands that my partner’s health is deteriorating and that the pet adjusts his behaviour accordingly. | |
| **Pleasure:**   - Owners often laugh and experience pleasure because of their pet. - A pet provides liveliness and a convivial atmosphere in the house. - Owners often take their pets on outings, for instance, when visiting friends or going on vacation. | | **Professional caregivers say:**   - I like to talk with clients about their pet. It is a simple topic of conversation, and it can help improve the atmosphere. - I support a client after their pet’s death. Due to the caregiving tasks and structure a pet provides, the pet has been a large and important part of the client’s life, - I see that a pet’s company is important to clients. | |
| **Grief:**   - The death of a pet can start an intense mourning process. - Owners often experience an emotional moment when they must put their pet down. - Owners sometimes organise a burial or cremation for their pet. - Owners place pictures in their home to commemorate a deceased pet. | |  |  |
| **N = 9** | **Content** | **Relevance** | **Formulation** |
| **Response to Feelings** | Item-CVI = 1.00 | Item-CVI = 1.00 | Item-CVI = 1.00 |
| **Emotional Support** | Item-CVI = 1.00 | Item-CVI = 1.00 | Item-CVI = 1.00 |
| **Pleasure** | Item-CVI = 1.00 | Item-CVI = 1.00 | Item-CVI = 1.00 |
| **Grief** | Item-CVI = .78 | Item-CVI = .67 | Item-CVI = 1.00 |
| **Emotional Aspects** | Scale-CVI = .94 | Scale-CVI = .92 | Scale-CVI = 1.00 |

*Note.* The statements are translated from Dutch.

**Appendix A6**

*Aspects of Caregiving*

| **Front: Themes** | | **Back: Stakeholder Perspectives** | |
| --- | --- | --- | --- |
| **Need of Caregiving:**   - Owners find pleasure in caring for their pet. - Owners enjoy spoiling their pet. - Owners often make arrangements with others in their network on pet care-related topics. | | **Clients say:**   - I like to take care of my pet and I notice that my pet takes care of me in a certain way. - I think it is nice to keep busy, and I have to take care of a lot of things thanks to my pet. - My pet’s health is the most important thing. - I worry about my pet. What will happen to my pet when I cannot care for my pet myself any longer? | |
| **Responsibility:**   - Owners believe that they must take care of their pet. - Owners feel as if they have the responsibility to make arrangements for their pet—for instance if they want to go away for a day. - Owners think that it is important to take responsibility for their pet—for instance when, because of an illness, they must put their pet down. | | **Family caregivers say:**   - I often help with the pet’s care. I walk and babysit the dog and go to the vet. - As my partner’s health deteriorates, I must take more responsibility to care for the pet. - I notice that the pet can become an obstacle. For example, I must arrange for someone to care for the pet if I want to go out for a day. | |
| **Sense of Safety:**   - Owners experience less stress because of their pet’s presence. - Pets provide their owners with a sense of safety and security. - Dogs bark and growl when there are unfamiliar people at the door. This can help owners feel safe. | | **Professional caregivers say:**   - When clients cannot care for their pet adequately anymore themselves, a pet can become a problem. - I will start a conversation with a client about the pet when I notice that it has become too difficult for a client to care for the pet. - I notice that clients and next of kin can react negatively to my advice on pet care, but they have the main responsibility. - Pets can influence healthcare-related decisions. Some clients postpone moving to a nursing home, or do not want to go to day care, because the pet will be alone. - I see that caring for a pet can give purpose and feel nice for clients. | |
| **Expenses:**   - Pets cost money. The costs can be divided into direct costs, like costs for food and the vet, and indirect costs, like a broken laptop because a dog sat on it. - Owners can experience costs as an obstacle when caring for a pet. | |  |  |
| **Worries:**   - Owners can worry about their pets—for example, about its health, possible death, or that it might run away. - Owners can also worry about having to leave their pet behind when moving to a nursing home or when the owner cannot independently care for the pet anymore. | |  |  |
| **N = 7** | **Content** | **Relevance** | **Formulation** |
| **Need of Caregiving** | Item-CVI = 1.00 | Item-CVI = 1.00 | Item-CVI = 1.00 |
| **Responsibility** | Item-CVI = .86 | Item-CVI = 1.00 | Item-CVI = 1.00 |
| **Sense of Safety** | Item-CVI = .71 | Item-CVI = .86 | Item-CVI = .86 |
| **Expenses** | Item-CVI = .71 | Item-CVI = .86 | Item-CVI = 1.00 |
| **Worries** | Item-CVI = 1.00 | Item-CVI = 1.00 | Item-CVI = 1.00 |
| **Aspects of Caregiving** | Scale-CVI = .86 | Scale-CVI = .94 | Scale-CVI = .97 |

*Note.* The statements are translated from Dutch.

**Appendix A7**

*Physical Health*

| **Front: Themes** | | **Back: Stakeholder Perspectives** | |
| --- | --- | --- | --- |
| **Exercise:**   - A pet motivates an owner to exercise more. - Taking care of, walking, or playing with a pet contributes to additional exercise. | | **Clients say:**   - I place my dove on my shoulder to help me relax. - Brushing my rabbit is my moment of meditation. - Walking and playing with my pet helps me exercise. I feel fitter. - I must walk the dog, even when I am not feeling well. - I would not go outside if I did not have my dog. | |
| **Routine:**   - Owners organise the day around pet-related tasks. - A pet indicates when it is hungry or wants to go out. - A pet can provide structure in the night-and-day rhythm of the owner. | | **Family caregivers say:**   - I feel fitter and more relaxed because I walk the dog. - I walk the dog together with my partner. | |
| **Relaxation:**   - Petting, caring for, the presence of, and talking to a pet can offer relaxation. - Walking the dog can be relaxing. | | **Professional caregivers say:**   - I use the pet as an instrument to motivate a client to exercise more. - I take a client’s structure regarding the pet into account in my daily planning. - I worry when a client has become unable to adequately care for a pet. - Clients with dementia can get lost when they walk the dog. - A pet provides structure due to the required caregiving; this can support clients with dementia. | |
| **Distraction from Discomfort:**   - A pet can distract its owner from discomfort or pain by seeking attention. | |  |  |
| **N = 12** | **Content** | **Relevance** | **Formulation** |
| **Exercise** | Item-CVI = .92 | Item-CVI = 1.00 | Item-CVI = 1.00 |
| **Structure** | Item-CVI = .92 | Item-CVI = 1.00 | Item-CVI = 1.00 |
| **Relaxation** | Item-CVI = 1.00 | Item-CVI = 1.00 | Item-CVI = 1.00 |
| **Distraction Discomfort** | Item-CVI = .92 | Item-CVI = .92 | Item-CVI = 1.00 |
| **Physical Health** | Scale-CVI = .94 | Scale-CVI = .98 | Scale-CVI = 1.00 |

*Note.* The statements are translated from Dutch.

**Appendix A8**

*Social Aspects*

| **Front: Themes** | | **Back: Stakeholder Perspectives** | |
| --- | --- | --- | --- |
| **Loneliness:**   - Owners feel less lonely because of their pet. - Owners do not come home to an “empty” house, the pet is always present. - Owners feel less lonely because they can talk to their pet. - Due to the pet’s presence, owners always have another living being around. | | **Clients say:**   - I like meeting people while walking the dog. - I tell (family) caregivers who I met while I was walking the dog. - The neighbour gives my dog treats; this helps with the contact. - My dog is an easy topic of conversation. - I talk with my pet, and this helps me feel less lonely. | |
| **Social facilitation:**   - A pet facilitates contact with others—for instance, when walking the dog. - A pet can serve as an “ice breaker” when there are visitors. - Another person helps with the pet’s care. This creates additional moments of contact with others. - Owners can become a member of a club—for example, a “bird club”—and this helps with additional social contacts. - A pet can help with social control; neighbours notice that the owner did not walk his dog and they may check up on him. | | **Family caregivers say:**   - I meet other people while walking the dog. - I talk with my partner about our pet. | |
|  |  | **Professional caregivers say:**   - I enjoy noticing that clients who own a pet are less lonely. - Clients who cannot walk the dog themselves do not benefit from additional social contacts. - When clients cannot take care of their pet adequately anymore, this may lead to neighbourhood nuisance—for instance, a barking dog. - I notice that the pet is a nice conversation topic, especially when I do not know what to say. - If I give the pet some attention, it makes it easier to connect to a client. | |
| **N = 10** | **Content** | **Relevance** | **Formulation** |
| **Loneliness** | Item-CVI = 1.00 | Item-CVI = 1.00 | Item-CVI = 1.00 |
| **Social Facilitation** | Item-CVI = 1.00 | Item-CVI = 1.00 | Item-CVI = 1.00 |
| **Social Aspects** | Scale-CVI = 1.00 | Scale-CVI = 1.00 | Scale-CVI = 1.00 |

*Note.* The statements are translated from Dutch.

**Appendix A9**

*Bidirectional Behaviour*

| **Front: Themes** | | **Back: Stakeholder Perspectives** | |
| --- | --- | --- | --- |
| **Physical Contact:**   - A pet seeks its owner’s attention. - Owners like to pet and cuddle with their pet, something pets also seem to enjoy. - Physical contact with their pets helps owners relax. - Owners view their pet as a conversation partner. | | **Clients say:**   - I talk to my pet, and it understands me. - I like it when my pet seeks my attention. - I like to cuddle with my pet. - I feel uneasy when my pet is not in my vicinity. - I take my pet everywhere. | |
| **Proximity:**   - Pets and owners seek each other’s proximity. - Owners feel loved and important when their pet seeks their proximity. - A pet does not leave their owner’s side. Even when the owner is ill. | | **Family caregivers say:**   - The dog likes to be in my and my partner’s vicinity. - When I comfort my partner the dog squeezes in between. - The pet is focussed more on my partner than on me. - The dog knows when my partner comes home and waits at the door. - I have entire conversations with the dog. | |
| **Responsiveness to Behaviour:**   - Pets notice changes in their surroundings. - Pets respond to their owner’s behaviour. - Owners often think that their pets understand them. | | **Professional caregivers say:**   - I notice that pets also seek my attention when I arrive at a client’s home. - Pets react differently when a client is in pain. - By the way a client handles a pet, I can tell that something is wrong—for instance, an arm that is hurting. - I notice that pets keep an eye on clients. - I see that cuddling with a pet does a lot for clients with dementia. | |
| **Mirroring:**   - The pet can serve as a mirror for their owners—for instance, owners notice that they are feeling tense through interaction with the pet. - Owners compare themselves with their pet— for instance, they both had surgery. | |  |  |
| **N = 13** | **Content** | **Relevance** | **Formulation** |
| **Physical Contact** | Item-CVI = 1.00 | Item-CVI = 1.00 | Item-CVI = .92 |
| **Proximity** | Item-CVI = 1.00 | Item-CVI = 1.00 | Item-CVI = 1.00 |
| **Response Behaviour** | Item-CVI = 1.00 | Item-CVI = 1.00 | Item-CVI = .92 |
| **Mirroring** | Item-CVI = .54 | Item-CVI = .62 | Item-CVI = .62 |
| **Bidirectional Behaviour** | Scale-CVI = .88 | Scale-CVI = .90 | Scale-CVI = .88 |

*Note.* The statements are translated from Dutch.
